# The effect of breakfast with different macronutrient composition on PYY, ghrelin, and ad libitum intake 4 h after breakfast in Indonesian obese women

**DOI:** 10.1186/s13104-018-3895-3

**Published:** 2018-11-03

**Authors:** Fiastuti Witjaksono, Widjaja Lukito, Andi Wijaya, Nagita Gianty Annisa, Joan Jutamulia, Fariz Nurwidya, Marcellus Simadibrata

**Affiliations:** 1 Department of Nutrition, Faculty of Medicine, Universitas Indonesia, Dr. Cipto Mangunkusumo Hospital, Jl. Salemba Raya No. 6, Jakarta, 10430 Indonesia; 2 Southeast Asian Ministers of Education Organization Regional Centre for Food and Nutrition (SEAMEO RECFON), Jl. Salemba Raya No. 6, Jakarta, 10430 Indonesia; 3 Department of Clinical Chemistry, Faculty of Medicine, Univeritas Hasanuddin, Jl. Perintis Kemerdekaan KM. 10, Makassar, 90245 Indonesia; 4 Department of Pulmonology and Respiratory Medicine, Faculty of Medicine, Universitas Indonesia, Persahabatan Hospital, Jalan Persahabatan Raya No.1, Jakarta, 13230 Indonesia; 5 Department of Internal Medicine, Faculty of Medicine, Universitas Indonesia, Dr. Cipto Mangunkusumo Hospital, Jl. Salemba Raya No. 6, 10430 Jakarta, Indonesia

**Keywords:** PYY, Ghrelin, Ad libitum intake, Protein, Obesity

## Abstract

**Objectives:**

Gut hormones, such as PYY and ghrelin, are associated with appetite control and obesity. Protein is thought to be the most satiating nutrient and could affect the production of several gut hormones. The purpose of the current study was to find the effect of breakfast with different protein composition on PYY, ghrelin, and ad libitum intake 4 h after breakfast.

**Results:**

This clinical trial involves 22 obese women participants. Subjects were given three types of breakfast: low protein consumption (12.4% protein), medium protein (23.5% protein), and high protein (40.6% protein). PYY and ghrelin levels were measured at 0, 15, 60, 120, and 180 min after breakfast. Ad libitum meal was given 4 h after breakfast and measured after. This study found that there is no significant difference in PYY and ghrelin level at each measurement time between different type of breakfast. This study also found no significant difference of ad libitum energy intake between different type of breakfast.

*Trial registration* ClinicalTrials.gov NCT03697486, 3 December 2018. Retrospectively registered

**Electronic supplementary material:**

The online version of this article (10.1186/s13104-018-3895-3) contains supplementary material, which is available to authorized users.

## Introduction

Obesity is one of the greatest health problems in the twenty first century. There are 1.6 billion adults currently classified as overweight, and 400 million are classified as obese. Among 105 different countries, except for the high-income countries, there was a greater overall prevalence of female obesity compared with male obesity [1]. Obesity has various adverse effects on health, including cardiovascular disease and type 2 diabetes mellitus. Several gut hormones have been associated with appetite control and obesity, including peptide YY (PYY) and ghrelin [2].

PYY is a peptide consisted of 36 amino acids, which is produced by L cells of the gastrointestinal tract. PYY functions as an appetite suppressing gut hormones. Circulating PYY increases satiety, inhibits gastrointestinal motility, inhibits pancreatic hormone secretion, and decreases food intake. Low levels of PYY have been associated with higher BMI and obesity [3]. Meanwhile, ghrelin is a 28 amino acid peptide that is expressed in many tissues, with the stomach is thought to be the source of circulating ghrelin in the body [4, 5]. Circulating ghrelin is associated with hunger and the level increases before a meal, and decreases post-prandially [6]. The postprandial release of PYY could also inhibit the release of ghrelin [7]. Obese patients have increased hunger and take longer to reach satiety. It is originally thought that the increase in hunger is caused by increased release of ghrelin [8]. However, ghrelin level is actually lower in obese patients. It is found that a greater number of calorie intake is needed for an obese person to produce a significant suppression in appetite than for a lean person. The magnitude of ghrelin suppression is also smaller in an obese person. This could cause a further problem in diet therapy of obese patients [9].

Macronutrient composition of a meal is associated with a different level of PYY and ghrelin secretion. In a study conducted in healthy men, a high protein breakfast decreases postprandial ghrelin production significantly compared to a high carbohydrate breakfast [10]. However, in a study done in patients with type 2 diabetes mellitus, there is no significant difference in postprandial ghrelin level between control diet (55% carbohydrate, 15% protein, 30% fat) compared to diet with higher protein and fat composition (30% carbohydrate, 30% protein, 40% fat) [11]. Postprandial PYY levels after high protein breakfast significantly increased as suggested in a study involving females with anorexia and bulimia nervosa [12]. Meanwhile, high carbohydrate meal elicits lowest PYY response. There are still ongoing debates about whether fat or protein elicits higher PYY response. Earlier research showed that fat produces higher PYY secretion, while more recent studies support protein as a greater stimulator of PYY. Weight status also influences the PYY response to each macronutrient [13, 14].

In this study, we hypothesized that breakfast with different macronutrient composition in obese subjects might implicates in the circulating ghrelin and PYY secretion. We also hypothesized that macronutrient composition in breakfast affects subject’s satiety, as determined by the ad libitum intake 4 h after breakfast.

## Main text

### Methods

The study is a double-blind randomized clinical trial with cross-over design for three interventions evaluating the effect of breakfast with different protein composition to several gut endocrines, level of satiety and ad libitum intake in obese patients. Two preliminary studies were done before the main study for taste-testing of the milk-based formula and to determine sample size. There are three interventions given, breakfast with low, medium, and high protein composition. All types of intervention are given to each participant in the study.

The inclusion criteria for this study is women aged 20–40 years old with BMI 25–30 (obese), with stable body weight within the last 6 months (body weight change less than 4 kg) and normal blood glucose level. The exclusion criteria are patients who never had breakfast, patients in weight loss program, patients consuming lipid-absorption inhibitor or appetite-suppressing drugs, and patients that have had gastrointestinal tract resection.

Before the intervention, participants were interviewed to obtain demographic data and food intake analysis. Participants were also given counseling in healthy dietary habit. At the day of intervention, participants should not eat within the last 10 h. Participants could drink mineral water up to 2 h before the intervention. Breakfast given is a milk-based formula which has to be finished within 15 min, and subjects could only consume 600 mL of mineral water within the next 4 h after intervention. Serum ghrelin and PYY level from participants were compared at each point of time (before intervention and 15, 60, 120, and 180 min after intervention). Calories from ad libitum intake in the different type of interventions were also compared.

Breakfast given is adjusted to participants’ energy requirement. Energy requirement is calculated by adding basal metabolic rate (BMR) with additional energy from physical activity around 40%. BMR is calculated by Cunningham equation. Breakfast was given as chocolate milk-based formula with volume 200 mL. The composition of the formula could be seen in Additional file 1: Table S1.

Ad libitum meal is a meal given that participants can eat as much as they wanted until they felt full. In this study, ad libitum meal is given 4 h after breakfast. The meal given composed of carbohydrates, protein, and fat. The meal prepared represented general subject’s taste and prepared in a way that would not decrease nor increase the subject’s appetite (not too delicious, not too spicy, salty, or sweet). Each food that was taken by participants would be weighed. After the participants done eating, the calorie in meal taken by participants was measured.

Statistical analysis was conducted using software SPSS version 20 (IBM Corporation, Chicago, IL, USA). Data distribution was determined by the Shapiro–Wilk test. Data analysis used the Friedman test because of non-normally distributed data. *P* value < 0.05 is considered to be significant.

### Results

There were 22 participants in this study. Sociodemographic characteristics of the participants are shown in Additional file 2: Table S2.

Mean ghrelin and PYY levels in subjects during 0, 15, 60, 120, and 180 min after each intervention can be seen in Additional file 3: Figure S1 and Additional file 4: Figure S2, respectively. Table 1 shows the comparison between ghrelin level in obese patients after intervention with low, medium, and high protein composition breakfast. Ghrelin level decreases until 60 min after intervention and started to increase after the 60 min (Additional file 3: Figure S1). Participants who were given high protein breakfast is shown to have a larger decline of ghrelin secretion while having a lower increase of ghrelin secretion 180 min after the intervention, although this was not statistically significant (Table 1).

**Table 1:** Comparison of ghrelin level after breakfast with different protein composition

Ghrelin level (pg/mL)	Low protein	Medium protein	High protein	P-value
Time after intervention				
0	219.90 (105.48–848)	270.35 (145.88–650)	248.53 (116–742)	0.786^F^
15	192.00 (81–879)	201.36 (59–621)	232.74 (100.71–526)	0.664^F^
60	181.07 (80.92–627)	202.89 (54–571)	176.67 (23–518)	0.057^F^
120	325.79 (90.96–871)	308.00 (155–643)	254.32 (88–605)	0.195^F^
180	414.50 (150.12–949)	355.00 (128–663)	321.05 (114–750)	0.094^F^

^F^ Friedman test

Meanwhile, PYY secretion increases with some fluctuations after the meal (Additional file 4: Figure S2). The highest level of PYY at 180 min was reached by participants who were given high protein breakfast. Table 2 shows the comparison between PYY level in obese patients after intervention with low, medium, and high protein composition breakfast. There was no significant difference in PYY level after breakfast with different protein composition.

**Table 2:** Comparison of PYY level after breakfast with different protein composition

PYY level (pg/mL)	Low protein	Medium protein	High protein	P-value
Time after intervention				
0	87.20 (19.9–253.1)	73.35 (19.9–323.3)	86.75 (19.9–238)	0.854^F^
15	93.35 (19.9–284.5)	77.65 (19.9–387.9)	96.90 (19.9–229.8)	0.640^F^
60	92.35 (19.9–354.6)	102.35 (19.9–427)	114.65 (19.9–358.1)	0.722^F^
120	102.45 (19.9–352.8)	115.10 (19.9–470.6)	120.85 (20.1–382)	0.700^F^
180	116.70 (23.7–305.1)	118.50 (19.9–377.7)	124.20 (19.9–377.9)	0.463^F^

^F^ Friedman test

To further test the hunger and satiety level after the intervention, we calculated energy intake from ad libitum meal. Mean of ad libitum intake after breakfast with different protein composition is shown in Additional file 5: Figure S3. We analyzed how many total, protein, fat, and carbohydrate calories were taken during an ad libitum meal. The comparison between ad libitum intake in obese patients after intervention with low, medium, and high protein composition breakfast is shown in Table 3. The highest calorie of ad libitum intake is found in participants after they were given low protein breakfast. However, the difference of total calories and calories from protein, fat, or, carbohydrate taken during ad libitum intake between each type of breakfast was not statistically significant.

**Table 3:** Comparison of ad libitum intake after breakfast with different protein composition

Ad libitum intake (kcal)	Low protein	Medium protein	High protein	P-value
Total	530.60 (423.4–757.3)	482.75 (365.2–979.8)	517.35 (311.1–797.1)	0.654^F^
Protein	100.40 (70–164.4)	94.20 (64.4–186)	94.20 (55.2–151.6)	0.261^F^
Fat	251.10 (166.5–404.1)	233.10 (160.2–543.6)	239.85 (142.2–442.8)	0.109^F^
Carbohydrate	179.60 (119.6–219.2)	178.20 (107.6–270.4)	163.80 (110.4–432)	0.635^F^

^F^ Friedman test

### Discussion

Appetite control is important in addressing the problem of obesity and is represented by the levels of PYY and ghrelin. Our study here investigated the potential implication of different protein content in the levels of hunter- and satiety-associated biomarkers.

There was a tendency that high protein breakfast results in larger decline of ghrelin secretion compared to low and medium protein, although it was not statistically significant. Most previous studies in lean and obese subjects found a significant difference in ghrelin secretion after high protein meal compared to high carbohydrate or high fat meal [10, 15].

This study also found that the PYY levels after breakfast with different protein composition was also not significantly different. Previous studies found that there is a significant difference in PYY secretion after high protein meal compared to high carbohydrate or high fat meal both in lean and obese subjects [15, 16].

The contrast between our study and previous studies regarding ghrelin and PYY level after breakfast with different macronutrient composition might be caused by the comparatively lower protein composition used in high protein meal, compared to previous studies. This study also has less striking difference in protein composition between low, medium, and high protein meal. A study of postprandial ghrelin response between control and higher protein diet with similar composition to our study also found that there is no significant difference in ghrelin secretion after two types of the meal [11].

There are conflicting results in the effect of different macronutrient composition to subsequent ad libitum energy intake. A study also found that there is no difference in subjective hunger/satiety ratings after eating variety of food rich in either carbohydrate or protein. Meanwhile, previous studies suggest that there is an attenuation and delay in hunger after high protein meal, with no difference in subsequent energy intake [17]. Another study found that there is no significant difference in ad libitum energy intake after meal with different macronutrient composition, even with a significant difference in gut hormone secretion [16]. Meanwhile, more recent study found that ad libitum energy intake was less following high protein meal compared to high carbohydrate meal [15].

Several studies have proposed that gastric volume and oral stimulation have more effect to satiety rather than the nutrient content of the food. This has been proven by experiment using gastric pyloric cuffs in rats [18]. The pathway might bypass the effect of gut hormones directly through central pathway. In one study, gastric volume expansion caused by gastric infusion of liquid activates areas in brain such as midbrain, amygdala, hypothalamus, and hippocampus. Meanwhile, oral stimulation activates hippocampus and anterior cingulate [19]. This findings might explain why even a significant difference in gut hormone secretion was not resulted in a significant difference in ad libitum energy intake. Our study also used the same volume and form of milk formula in each type of intervention, causing same gastric volume expansion after each type of intervention.

### Conclusion

This study found no significant difference in ghrelin and PYY level after breakfast with low, medium, and high protein composition. There was also no significant difference in ad libitum energy intake after breakfast with different protein composition. Further studies are needed to find an optimal diet for obese patients that capable of increasing satiety and reducing subsequent food intake in order to reduce their weight more comfortably.

## Limitations

This study only involved female participants which limits the generalizability in the wider population. Data distribution in this study was not normal, which led us to use nonparametric test. Other limitation is the difference of protein composition between groups being too small to show the effect on ghrelin and PYY levels therefore future study with higher degree of difference in protein amount would be important to clarify the role of protein composition in Indonesian subjects with obesity.

## Additional files

**Additional file 1: Table S1.** Macronutrient composition in breakfast formula. Calorie, protein, carbohydrate, fat, fiber, form, volume, density and flavour of the high protein breakfast formula, medium protein breakfast formula, and low protein breakfast formula.

**Additional file 2: Table S2.** Sociodemographic characteristics of subjects. Characteristics of the subjects, such as age, education, body mass index, income category, and teenage nutritional status.

**Additional file 3: Figure S1.** Mean ghrelin level in subjects after intervention. Mean levels of ghrelin in 0, 15, 60, 120, and 180 min after the intervention.

**Additional file 4: Figure S2.** Mean PYY level in subjects after intervention. Mean levels of PYY in 0, 15, 60, 120, and 180 min after the intervention.

**Additional file 5: Figure S3.** Mean ad libitum intake in subjects 4 h after breakfast. Mean ad libitum intake in 0, 15, 60, 120, and 180 min after the intervention.
