# Supplementary material for: The effect of breakfast with different macronutrient composition on PYY, ghrelin, and ad libitum intake 4 h after breakfast in Indonesian obese women
Source: BMC Res Notes. 2018 Nov 3;11:787. doi: 10.1186/s13104-018-3895-3 (PMC6215622; doi:10.1186/s13104-018-3895-3)
Supplement: Supplementary file 1 — Additional file 1: Table S1. Macronutrient composition in breakfast formula. Calorie, protein, carbohydrate, fat, fiber, form, volume, density and flavour of the high protein breakfast formula, medium protein breakfast formula, and low protein breakfast formula. [file 13104_2018_3895_MOESM1_ESM.docx]

**Table S1. Macronutrient Composition in Breakfast Formula**

| Breakfast | High Protein | % | Medium protein | % | Low protein | % |
| --- | --- | --- | --- | --- | --- | --- |
| Calorie | 202.68 |  | 204.23 |  | 200.85 |  |
| Protein (g) | 20.59 | 40.6 | 12.04 | 23.5 | 6.25 | 12.4 |
| Carbohydrate (g) | 20.39 | 40.2 | 29.08 | 56.9 | 34.27 | 68.2 |
| Fat (g) | 5.05 | 22.4 | 5.17 | 22.7 | 5.06 | 22.6 |
| Fiber (g) | 2.25 |  | 2.25 |  | 2.25 |  |
| Form | Liquid |  | Liquid |  | Liquid |  |
| Volume | 200 mL |  | 200 mL |  | 200 mL |  |
| Density | 1 cal/1 mL |  | 1 cal/1 mL |  | 1cal/1mL |  |
| Flavor | Chocolate |  | Chocolate |  | Chocolate |  |
