# Supplementary material for: The effect of breakfast with different macronutrient composition on PYY, ghrelin, and ad libitum intake 4 h after breakfast in Indonesian obese women
Source: BMC Res Notes. 2018 Nov 3;11:787. doi: 10.1186/s13104-018-3895-3 (PMC6215622; doi:10.1186/s13104-018-3895-3)
Supplement: Supplementary file 2 — Additional file 2: Table S2. Sociodemographic characteristics of subjects. Characteristics of the subjects, such as age, education, body mass index, income category, and teenage nutritional status. [file 13104_2018_3895_MOESM2_ESM.docx]

**Table S2. Sociodemographic Characteristics of Subjects**

| Characteristics | Subjects (n=22) |
| --- | --- |
| Age | 31.41±5.29 |
| Education (n,%)  Low  High | 0 (0)  22 (100) |
| Body Mass Index (BMI) | 28.59±2.35 |
| Income (n,%)  < Provincial minimum wage  > Provincial minimum wage | 0 (0)  22 (100) |
| Teenage Nutritional Satus (n,%)  Thin  Normal  Overweight | 3 (13.6)  14 (63.7)  5 (22.7) |
