# Supplementary figures and images for: The effect of breakfast with different macronutrient composition on PYY, ghrelin, and ad libitum intake 4 h after breakfast in Indonesian obese women
Source: BMC Res Notes. 2018 Nov 3;11:787. doi: 10.1186/s13104-018-3895-3 (PMC6215622; doi:10.1186/s13104-018-3895-3)

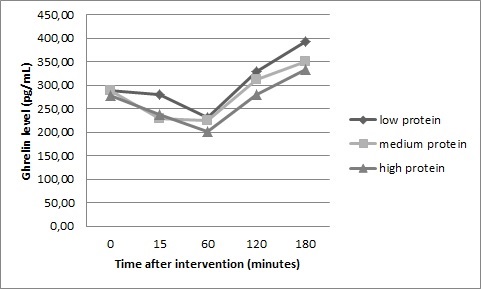

Supplement: Supplementary file 3 — Additional file 3: Figure S1. Mean ghrelin level in subjects after intervention. Mean levels of ghrelin in 0, 15, 60, 120, and 180 min after the intervention. [file 13104_2018_3895_MOESM3_ESM.jpg]

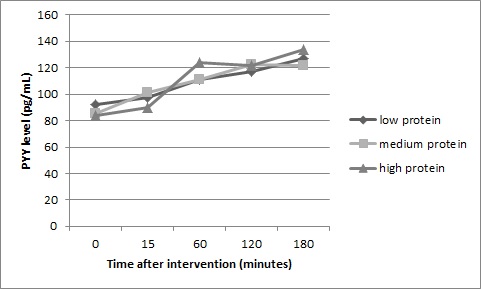

Supplement: Supplementary file 4 — Additional file 4: Figure S2. Mean PYY level in subjects after intervention. Mean levels of PYY in 0, 15, 60, 120, and 180 min after the intervention. [file 13104_2018_3895_MOESM4_ESM.jpg]

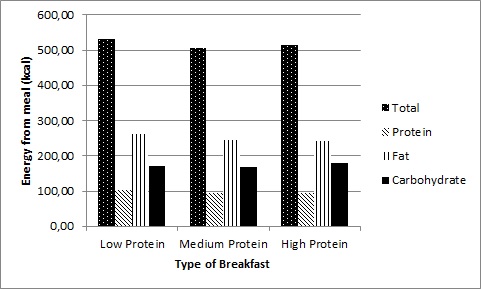

Supplement: Supplementary file 5 — Additional file 5: Figure S3. Mean ad libitum intake in subjects 4 h after breakfast. Mean ad libitum intake in 0, 15, 60, 120, and 180 min after the intervention. [file 13104_2018_3895_MOESM5_ESM.jpg]
